# Supplementary material for: Neoadjuvant Systemic Treatment of Primary Angiosarcoma
Source: Cancers (Basel). 2020 Aug 12;12(8):2251. doi: 10.3390/cancers12082251 (PMC7464310; doi:10.3390/cancers12082251)
Supplement: Supplementary file 1 [file cancers-12-02251-s001.pdf]

Table S1. A quality assessment of the included cohort studies using the Newcastle-Ottawa scale for cohort studies [47].

Cohort study	Selection	Comparability	Outcome
Guadagnolo 2011	*****	**	**
Oxenberg 2015	****	**	**
Sinnamon 2016	****	*	***
Li 2016	***	*	**
Abu Saleh 2017	****	**	***
Fayette 2007	***	-	***
